# A neural network multi-task learning approach to biomedical named entity recognition

**DOI:** 10.1186/s12859-017-1776-8

**Published:** 2017-08-15

**Authors:** Gamal Crichton, Sampo Pyysalo, Billy Chiu, Anna Korhonen

**Affiliations:** Language Technology Laboratory, DTAL, University of Cambridge, 9 West Road, Cambridge, CB39DB UK

**Keywords:** Multi-task learning, Convolutional neural networks, Named entity recognition, Biomedical text mining

## Abstract

**Background:**

Named Entity Recognition (NER) is a key task in biomedical text mining. Accurate NER systems require task-specific, manually-annotated datasets, which are expensive to develop and thus limited in size. Since such datasets contain related but different information, an interesting question is whether it might be possible to use them together to improve NER performance. To investigate this, we develop supervised, multi-task, convolutional neural network models and apply them to a large number of varied existing biomedical named entity datasets. Additionally, we investigated the effect of dataset size on performance in both single- and multi-task settings.

**Results:**

We present a single-task model for NER, a Multi-output multi-task model and a Dependent multi-task model. We apply the three models to 15 biomedical datasets containing multiple named entities including Anatomy, Chemical, Disease, Gene/Protein and Species. Each dataset represent a task. The results from the single-task model and the multi-task models are then compared for evidence of benefits from Multi-task Learning.

With the Multi-output multi-task model we observed an average F-score improvement of 0.8% when compared to the single-task model from an average baseline of 78.4%. Although there was a significant drop in performance on one dataset, performance improves significantly for five datasets by up to 6.3%. For the Dependent multi-task model we observed an average improvement of 0.4% when compared to the single-task model. There were no significant drops in performance on any dataset, and performance improves significantly for six datasets by up to 1.1%.

The dataset size experiments found that as dataset size decreased, the multi-output model’s performance increased compared to the single-task model’s. Using 50, 25 and 10% of the training data resulted in an average drop of approximately 3.4, 8 and 16.7% respectively for the single-task model but approximately 0.2, 3.0 and 9.8% for the multi-task model.

**Conclusions:**

Our results show that, on average, the multi-task models produced better NER results than the single-task models trained on a single NER dataset. We also found that Multi-task Learning is beneficial for small datasets. Across the various settings the improvements are significant, demonstrating the benefit of Multi-task Learning for this task.

**Electronic supplementary material:**

The online version of this article (doi:10.1186/s12859-017-1776-8) contains supplementary material, which is available to authorized users.

## Background

Biomedical text mining and Natural Language Processing (NLP) have made tremendous progress over the past decades, and are now used to support practical tasks such as literature curation, literature review and semantic enrichment of networks [1]. While this is a promising development, many real-life tasks in biomedicine would benefit from further improvements in the accuracy of text mining systems.

The necessary first step in processing literature for biomedical text mining is identifying relevant named entities such as protein names in text. This task is termed Named Entity Recognition (NER). High accuracy NER systems require manually annotated named entity datasets for training and evaluation. Many such datasets have been created and made publicly available. These include annotations for a variety of named entities such as genes and proteins [2], chemicals [3] and species [4] names. Because manual annotations are expensive to develop, datasets are limited in size and not available for many sub-domains of biomedicine [5, 6]. As a consequence, many NER systems suffer from poor performance [7, 8].

The question of how to improve the performance of NER, especially in the very common situation where only limited annotations are available, is still an open area of research. One potentially promising solution is to use multiple annotated datasets together to train a model for improved performance on a single dataset. This can help since datasets may contain complementary information that can help to solve individual tasks more accurately when trained jointly.

In machine learning, this approach is called *Multi-task Learning* (MTL) [9]. The basic idea of MTL is to learn a problem together with other related problems at the same time, using a shared representation. When tasks have commonality and especially when training data for them are limited, MTL can lead to better performance than a model trained on only a single dataset, allowing the learner to capitalise on the commonality among the tasks. This has been previously demonstrated in several learning scenarios in bioinformatics and in several other application areas of machine learning [10–12].

A variety of different methods have been used for MTL, including neural networks, joint inference, and learning low dimensional features that can be transferred to different tasks [11, 13, 14]. Recently, there have been exciting results using Convolutional Neural Networks (CNNs) for MTL and transfer learning in image processing [15] and NLP [16–18], among other areas.

In this work, we investigate whether a MTL modeling framework implemented with CNNs can be applied to biomedical NER to benefit this key task. This is, to the best of our knowledge, the first application of this MTL framework to the task. Like other language processing tasks in biomedicine, NER is made challenging by the nature of biomedical texts, e.g. heavy use of terminology, complex co-referential links, and complex mapping from syntax to semantics. Additionally, the annotated datasets available vary greatly in the nature of named entities (e.g. species vs. disease), the granularity of annotation, as well as in the specific domains they focus on (e.g. chemistry vs. anatomy). It is therefore an open question whether this task can benefit from MTL.

Due to the aforementioned disparities between data-sets, we treat each dataset as a separate task even when the annotators sought to annotate the same named entities. Thus datasets and tasks are used interchangeably. We first develop a single task CNN model for NER and then two variants of a multi-task CNN. We apply these to 15 datasets containing multiple named entities including Anatomy, Chemical, Disease, Gene/Protein and Species. The results are then compared for evidence of benefits from MTL. On one MTL model we observe an average F-score improvement of 0.8% with a range of –2.4 to 6.3% on MTL in comparison with single task learning from an average baseline F-score of 78.4% with range 68.6 to 83.9%. Although there is a significant drop in performance on one dataset, performance improves significantly for five datasets. For the other MTL model we observe an average F-score improvement of 0.4% with a range of –0.2 to 1.1% on MTL in comparison with single task learning from the same baseline. There is no significant drop in performance on any dataset, and performance improves significantly for six datasets. These are promising results which show the potential of MTL for biomedical NER.

The “Motivation” section explains the motivations behind this work and how it can contribute to biomedical text mining. The “Related work” section describes the background and related work in MTL and NER. Details of the models, methods and datasets used are in the “Methods” section. Our experiments are detailed in the “Experiments” section. We analyse the results and their implications in the “Results and discussion” section. The “Conclusion” section concludes the presented work and explains possible future directions.

### Motivation

Previous work have demonstrated the benefits of MTL. These include leveraging the information contained in the training signals of related tasks during training to perform better at a given task, combining data across tasks when few data are available per task and discovering relatedness among data previously thought to be unrelated [12, 17, 19]. These benefits can be seen in potentially ambiguous terms which are spelled the same and are named entities in some situations, but not in others. Some training sets may contain examples of both so that a model can learn to distinguish between them, but others may only contain one type. A model trained with a dataset combination which contains both types (even if each dataset contains only one but they are opposites) can learn to distinguish between them and perform better.

We are similarly interested in these benefits, but are additionally interested in the following benefits, given the particular challenges of biomedical text mining.

#### Making the best use of information in existing datasets

Given the level of knowledge interaction and overlap in the biomedical domain, it is conceivable that signals learned from one dataset could be helpful in learning to perform well on other datasets. As an example, two of the Gene/Protein datasets we used contain *Pebp2* (and its variants) in their evaluation data but not in their training data. There are three other datasets which do contain *Pebp2* (and its variants) in their training data so models trained with these datasets may do better on the evaluation than models trained in isolation. If a model can utilize such information it could conceivably perform better as a result of having access to this additional knowledge. Currently, when models use additional knowledge as guidance it is typically handcrafted and passed to models during training rather than learned as part of the training process.

#### Efficient creation and use of datasets

The datasets used to train supervised and semi-supervised models are expensive to create. They typically contain manual annotations by highly trained domain specialists (e.g. biologists with sufficient linguistics training) often covering thousands of instances (e.g. of named entities or relations) each. If models which facilitate the transfer of knowledge between existing datasets can be developed and understood, they may be able to reduce the annotation overhead. For example, such models may be able to detect which type of annotations are really needed and which are not because the information is already included in another dataset or the knowledge requirements of tasks overlap. This can help to focus annotation efforts aimed at types not covered in any existing datasets and can aid in obtaining required annotations faster even if the resulting datasets are smaller. Caruana [9] demonstrated that *sampling data amplification* can help small datasets in MTL where tasks are related by combining the estimates of the learned parameters to obtain better estimates than it would by estimating them from small samples which may not provide enough information for modeling complex relationships between input and predictions.

It can be tempting to think that these objectives can be met by simply combining the existing corpora into a single large corpus which can then be used to train a model. The work of [20], which investigated the feasibility of this for gene/protein named entities in three datasets, showed otherwise. They found that simply using combined data resulted in performance drops of nearly 12% F-score and identified as the main cause of the drop incompatibilities in the annotations due to the fact that they were made by different groups with no explicit consensus about what should be annotated.

Thus the problem of utilizing all the knowledge in existing datasets in a single model to gain the benefits of doing so, including those highlighted in this section, remains a challenging open problem in biomedical NLP.

### Related work

MTL uses inductive transfer in such a way as to improve learning for a task by using signals of related tasks discovered during training. The work of [9] motivated and laid the foundation for much of the work done in MTL by demonstrating feasibility and important early findings. The author applied MTL on various detailed synthetic and four real-world problems. He highlighted the importance of the tasks being related and defined to a great extent what *related* meant in the context of MTL. He defines a related task as one which gives the main task better performance than when it is trained on its own. He found that: related tasks are not correlated tasks, related tasks must share input features and hidden units to benefit each other during training and finally that related tasks would not always help each other. This final finding may seem at odds to the given definition of related, but he explains that the learning algorithm also affects whether related tasks are able to benefit each other and allows for the existence of related tasks which the algorithm may not be able to take advantage of.

Since then, there have been work which like this one used MTL for NLP tasks though on general domain data. Collobert et al. [16] sought to use MTL in a unified model to gain increased performance in several core NLP tasks: NER, chunking, Part of Speech (POS) tagging and semantic role labeling with neural networks. They achieved a unified model which performed all tasks without significant degradation of performance, but there was little benefit from MTL. Ando and Zhang [11] investigated learning functions which serve as good predictors of good classifiers on hypothesis spaces using MTL of labeled and unlabeled data. They reported good results when tested on several machine learning tasks including NER, POS tagging and hand-written digit image classification. Liu et al. [21] used multi-task deep neural networks to learn representations for information retrieval and semantic classification by jointly training a model for both tasks which has shared and private layers. Their model outperformed strong baselines for both query classification and web search tasks. MTL can be related in some sense to joint learning and to that end [22] presented a model which used single-task annotated data as additional information to improve the performance of a model for jointly learning two tasks over five datasets.

MTL has also been applied in the biomedical domain to improve results in Text Mining and NLP. Qi et al. [23] used semi-supervised MTL to classify whether protein pairs were interacting. They first trained a model on supervised classification task with fully-labeled examples then shared some layers of the model with a semi-supervised model which is trained on only partially-labeled examples. Qi et al. [24] used MTL for small interfering RNA (siRNA) efficiency prediction by learning several functions of efficiency indicators which gave a predictor for siRNA efficiency. In [25] the authors used multi-task learning to predict a range of Mental Health conditions from users’ tweets by using demographic attributes and mental states as multiple tasks to feed-forward neural networks.

MTL’s use in the biomedical domain has also been seen in image classification where CNNs, the model we use, is more prevalent. Zeng and Ji [15] successfully used the weights of CNNs from [26] trained on general domain images as the starting point for further training on images in the biomedical domain to gain improved performance. Zhang et al. [27] used MTL methods with CNNs and labeled images to fine-tune models trained on natural images to extract features for specific biomedical tasks. Their features learned from deep models with multi-task methods outperformed other methods in annotating gene expression patterns.

In summary, research in MTL using neural networks has produced a wide spectrum of approaches. These approaches have yielded impressive results on some tasks (e.g. image processing) while results on others (e.g. mainstream NLP) have been more modest. We apply MTL to a NLP task and on a scale where it could be highly beneficial but where it has not been investigated yet: biomedical NER across 15 datasets. We present a single task and two multi-task models which train these datasets and compare their performance across the two settings. We were able to achieve significant gains in several datasets with both of the multi-task models despite the difference in the way in which they apply MTL.

## Methods

### Pre-trained biomedical word embeddings

All our experiments used pre-trained, static word representations as input to the models. These representations are called *word embeddings* and are the inputs to most current neural network models which operate on text. Popular embeddings include those created by [28, 29]. Those are however aimed at general domain work and can produce very high out-of-vocabulary rates when used on biomedical texts, thus for this work we used the embeddings created in [30] which are created from biomedical texts. An embedding for unknown words was also trained for use with out-of-vocabulary words during training of our models.

### Datasets

We used 16 biomedical corpora: 15 focused on biomedical NER and one on biomedical POS tagging. POS tagging is a sequential labeling task which assigns a part-of-speech (e.g. Verb, Nouns) to each word in text. We chose datasets which were publicly available and included sufficient amounts of the most utilized named entities in bioinformatics: Anatomy, Chemical, Disease, Gene/Protein and Species. The names of the datasets and information about their corresponding named entities are listed in Table 1. Details of their creation, prior use, and comparison of the original data to the versions we prepared for sequential labeling can be found in Additional file 1 provided on the paper’s Github page which is https://github.com/cambridgeltl/MTL-Bioinformatics-2016.

**Table 1:** The datasets and details of their annotations

Dataset	Contents	Entity counts
AnatEM [38]	Anatomy NE	13,701
BC2GM [2]	Gene/Protein NE	24,583
BC4CHEMD [3]	Chemical NE	84,310
BC5CDR [5]	Chemical, Disease NEs	Chemical: 15,935; Disease:12,852
BioNLP09 [52]	Gene/Protein NE	14,963
BioNLP11EPI [53]	Gene/Protein NE	15,811
BioNLP11ID [53]	4 NEs	Gene/Protein: 6551; Organism: 3471;
		Chemical: 973; Regulon-operon: 87
BioNLP13CG [54]	16 NEs	Gene/Protein: 7908; Cell: 3492; Cancer: 2582
		Chemical: 2270; Organism: 1715; Multi-tissue structure: 857;
		Tissue: 587; Cellular component: 569; Organ: 421;
		Organism substance: 283; Pathological formation: 228; Amino acid: 135;
		Immaterial anatomical entity: 102; Organism subdivision: 98;
		Anatomical system: 41; Developing anatomical structure: 35
BioNLP13GE [55]	Gene/Protein NE	12,057
BioNLP13PC [56]	4 NEs	Gene/Protein: 10,891; Chemical: 2487;
		Complex: 1502; Cellular component: 1013
CRAFT [57]	6 NEs	SO: 18,974; Gene/Protein: 16,064;
		Taxonomy: 6868; Chemical: 6053; CL: 5495; GO-CC: 4180
Ex-PTM [58]	Gene/Protein NE	4698
JNLPBA [44]	5 NEs	Gene/Protein: 35,336; DNA: 10,589; Cell Type: 8639
		Cell Line: 4330; RNA: 1069
Linnaeus [4]	Species NE	4263
NCBI-Disease [6]	Disease NE	6881
GENIA-PoS [59]	PoS-Tagging	N/A

A point of concern for our method would be whether there is significant overlap between the training sentences of one dataset and the test sentences in another as this would expose the model to examples which it would be evaluated on. We found that the test sets for BC5CDR and BioNLP09 overlapped with the BC2GM train sets 0.02 and 0.37%, respectively, and that the test set for JNLPBA overlapped with 0.08% of the BioNLP09 train set. These figures were not deemed large enough to influence the validity of the experiments so no steps were taken to resolve them.

### Experimental setting

We first trained a single-task model for each of the datasets in multiple settings then trained them in several MTL settings. The results of the performance in the multi-task settings were compared to those in similar single-task settings. The multi-task settings are detailed in the “Experiments” section and involved two multi-task models which we will introduce in this section while the others involved variations on subsets of the datasets trained jointly and variation in dataset sizes.

At each training step a fixed amount of training examples (mini-batch) from the dataset being trained was selected after shuffling the training examples. For the multi-task models this mini-batch would be randomly selected from one of the datasets being trained and the model trained with only the part of the model relevant to the selected dataset activated.

Our models were trained to perform NER as a sequential tagging task where each word in a sentence is tagged with an appropriate tag. The tags used were *Single-named entity*, *Begin-named entity*, *In-named entity*, *End-named entity* and *Out* where *named entity* differed according to the type of named entities in the dataset (gene/proteins, chemicals etc.). A word is tagged *Single-named entity* if it is the only word in the named entity, while entities of two or more words begin with *Begin-named entity* and end with *End-named entity*. *In-named entity* is used for words which occur between *Begin-named entity* and *End-named entity* tags if a named entity has three or more words. *Out* is used if a word is not a part of any named entity. Each dataset contained train, development and test sections and a split into these sections was introduced if none existed. Models were trained on the train section, their hyperparameters were tuned on the development section and the final evaluations were done on the test section.

The three main models in this work are all CNNs with varying architectures, and a feed-forward model was used as a baseline. The models and relevant method details are described in this section. We treated each dataset as a separate task. The details of the datasets used and their respective annotation information are listed in Table 1.

The input layer of all the models accept representations of the focus word to be classified and a context of *n* words before and after it to give a total of 2n + 1 words. The representations remain unchanged during training. During pre-processing, special tokens representing sentence breaks are added. The Viterbi algorithm used for calculating binary transition probabilities as by [31] is applied to the outputs of all models. An overview of this is as follows, first a binary transition matrix is calculated from the training data labels where for each possible tag transition sequence a score of 1 is given if the training data contains the transition and 0 if such a transition does not exist. The information in this matrix is then applied to the sequence of predicted tags and used to update any predicted tag sequences which are not seen in the training data (i.e. with tag transition score 0) with a tag transition sequence which was seen.

#### Baseline model

This was a feed-forward neural network with a hidden Rectified Linear Unit (ReLU) [32] activation layer leading to an output layer with Softmax activation.

#### Single task model

**Fig. 1:**
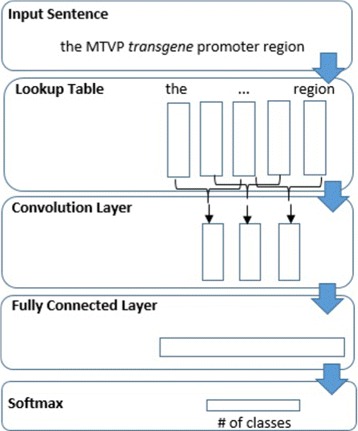
Single-task convolutional model

The input layer leads to a convolutional layer which applies multiple filter sizes to a window of words in the input in a single direction. To apply each filter in only a single direction over the window of words, the width of the filter always equals the amount of dimensions of the word embeddings. The outputs of all filters then go to a layer with ReLU activation. We concatenate and reshape the outputs before they pass into a fully connected layer then an output layer with a Softmax activation which classifies the focus word by selecting the label with the maximum value of the Softmax output. This model is similar to the one used by [17] but there is no max-pooling after the convolution layer. We refrain from using pooling layers so that positional information in the input would not be lost. We experimented with max-pooling and found that performance improved when it was not used. See Fig. 1 for a depiction of this model.

#### Multi-output multi-task model

**Fig. 2:**
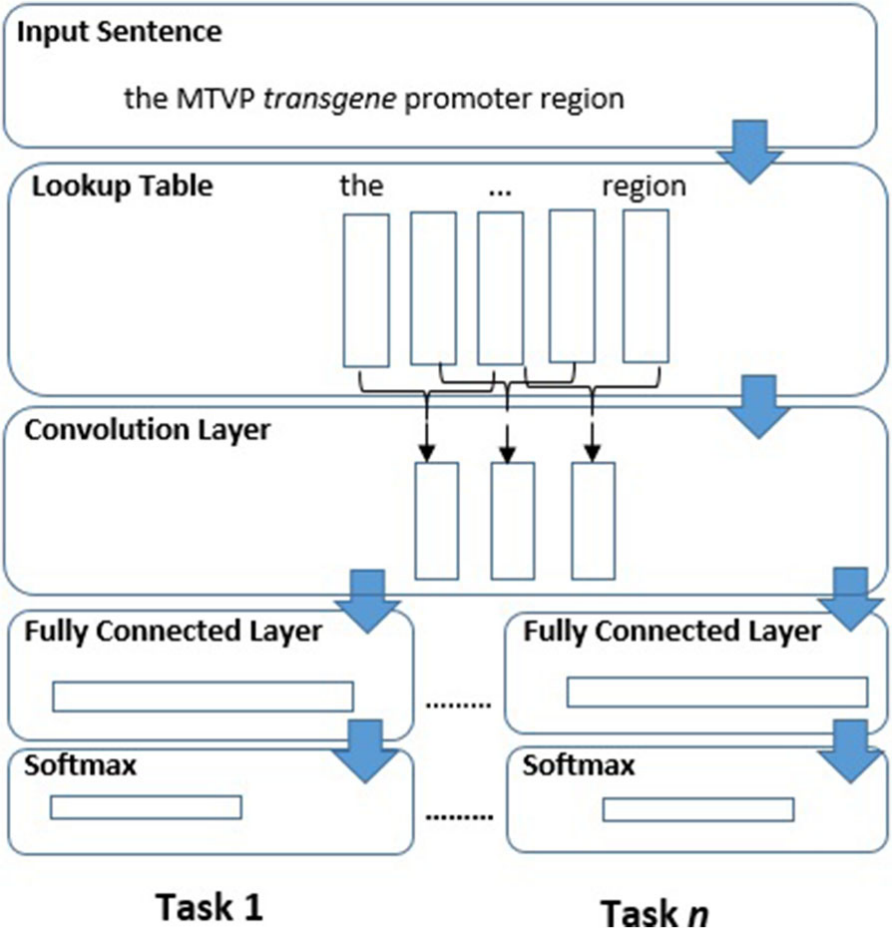
Multi-output multi-task convolutional model

The first multi-task model is similar to the single-output model described in the “Single task model” section up to the output layer. In this model there are separate output layers for each task the model learns. Thus a private output layer with Softmax activation represents each task but all tasks share the rest of the model. This model is similar to the one used by [16] but there are convolutional layers. It is also similar to the one used by [17] but we share the convolution layers in addition to the word embeddings and there is again no max-pooling. Figure 2 depicts this model.

#### Dependent multi-task model

**Fig. 3:**
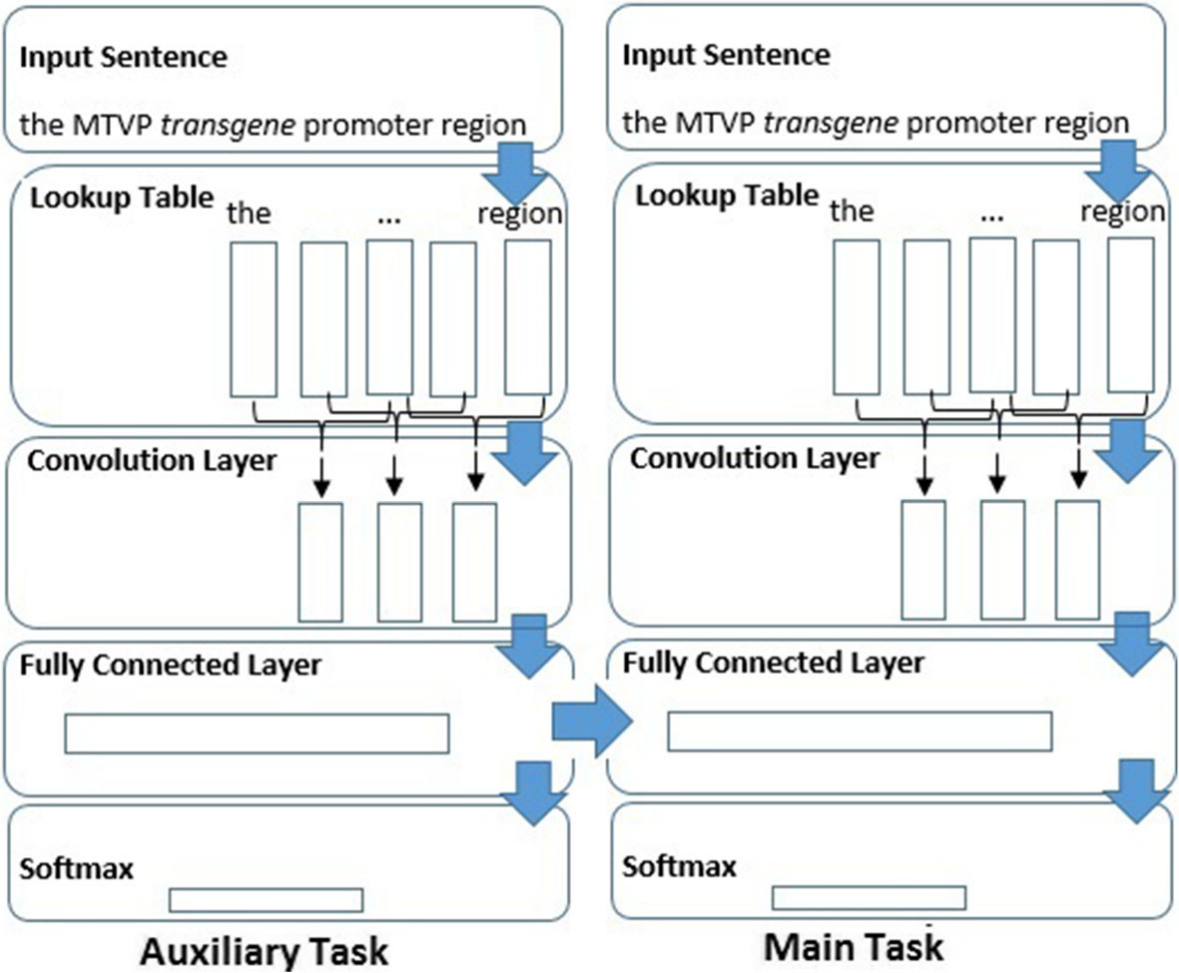
Multi-task dependent convolutional model

This model makes use of the fact that some NLP tasks are able to use information from other tasks to perform better. An example of this is that NER may utilize the information contained in the output of POS tagging to improve its performance. This model combines two of the single-task models described in the “Single task model” section with one model accepting input from the other. The first model trains for the auxiliary task (POS tagging in our example), then that trained model is used in the training of the second part of the model for the main task (NER in our example) by concatenating the fully connected layers of the model trained for the auxiliary task and the one trained for the main task. The use of this arrangement is similar to the one used by [33] but our layers between word embeddings and Softmax are convolutions and fully-connected layers. See Fig. 3 for a depiction of this model.

### Experiments

All inputs consisted of a focus word and three words to the left and right of it to give a seven word context window. The baseline model had one hidden layer of size 300 and was trained with the Stochastic Gradient Descent optimizer using mini-batch size 50. All CNN models used dropout [34] with a probability of 0.75 at the fully connected layer only. No other form of regularization was used. The CNN models used 100 filters of sizes of 3, 4 and 5 and a learning rate of 10^−4^ was used with the Adam [35] optimizer on mini-batch size 200. The loss function used was Categorical Crossentropy. These settings were chosen as they produced the best results from parameter tuning on the development sections of BC2GM, BioNLP09, BC5CDR and AnatEM.

Each dataset was used to train a single-task model (“Single task model” section). Details of these as well as the various multi-task experiments utilizing multi-task models (“Multi-output multi-task model” and “Dependent multi-task model” sections) follow.

**Baseline experiments:** We completed tests with the baseline model using each of the datasets listed in Table 1.

**Effect of datasets on each other:** To determine the exact effect that each NER dataset had on every other one, the multi-task model described in the “Multi-output multi-task model” section was used to train each NER dataset with every other one. That is, a Multi-output multi-task model was trained for each ordered combination of the datasets to give 15 × 14 models.

**Grouping datasets with similar named entities:** Several datasets in Table 1 sought to annotate the same named entities (Chemical, Cell, Cellular Component, Disease, Gene/Protein, Species). We created modified versions of these datasets which extracted only those entity annotations and then grouped the datasets which annotated the same named entity. This was done by changing the labels of the classes of annotations of entities, other than the one in focus, to the ‘Out’ class. These groups were used to train the Multi-output multi-task model from the “Multi-output multi-task model” section.

**Multi-task experiments with complete dataset suite:** The first part of this experiment used all the NER datasets to train the Multi-output multi-task model (“Multi-output multi-task model” section). In the second part, the Dependent multi-task model (“Dependent multi-task model” section) was used to train each dataset with the GENIA-PoS dataset as the auxiliary task.

**Correlation of dataset size and effect of Multi-task Learning:** To determine how the effect of Multi-task Learning varies with dataset size for our chosen datasets, we used only 50, 25 and 10% of the training section of each dataset in both single and multi-task settings and observed the effect this had on performance. In the multi-task settings, the reduced dataset was trained only with the dataset which best improved it as determined from the effects experiment described above (i.e. the dataset listed in the ‘Best Dataset’ column of Table 2). The Multi-output multi-task model (“Multi-output multi-task model” section) was used for these experiments.

**Table 2:** Best positive effects

Dataset	STM	Best MO-MTM	Best dataset
AnatEM	81.55	**81.68**	NCBI-Disease
BC2GM	**72.63**	72.21	Ex-PTM
BC4CHEMD	**82.95**	80.31	BioNLP13GE
BC5CDR	83.66	**83.77**	BioNLP11EPI
BioNLP09	83.90	**84.16**	BioNLP13GE
BioNLP11EPI	77.72	**78.10**	BioNLP09
BioNLP11ID	81.50	82.26*	BioNLP13GE
BioNLP13CG	76.74	77.33*	BioNLP13PC
BioNLP13GE	73.28	76.09*	BioNLP11EPI
BioNLP13PC	80.61	**80.94**	Ex-PTM
CRAFT	**79.55**	78.48	BioNLP13GE
Ex-PTM	68.56	73.58*	BioNLP11EPI
JNLPBA	**69.60**	68.92	BioNLP13GE
Linnaeus	**83.98**	83.63	NCBI-Disease
NCBI-Disease	80.26	**80.74**	Ex-PTM
Average	78.43	78.81	N/A

Datasets in rightmost column are the auxiliary ones. (**Bold**: best scores, *: statistically significant)

## Results and discussion

In the tables of results, columns headed STM refer to results from the single-task model (“Single task model” section), columns headed MO-MTM refer to results from the Multi-output multi-task model (“Multi-output multi-task model” section) and columns headed D-MTM refer to the Dependent multi-task model (“Dependent multi-task model” section). The scores reported are macro F1-Scores (a single precision and recall calculated for all types) of the entities at the mention level so exact matches are required for multi-word entities. Best results are shown in bold and statistically significant score changes are shown with an asterisk. All statistical tests were done using a two-tailed *t*-test with *α* = 0.05. The accuracy on the POS tagging task for the model used in the Dependent multi-task model training was 98.10%.

### Multi-task learning effect of each dataset

Information about the maximum scores achieved for each dataset is shown in Table 2. In 4 of the 15 datasets, there were maximums which were significantly higher than the single-task maximum scores shown in the ‘STM’ column of the table. This illustrates that for these datasets there is at least one other dataset in our suite which could be trained jointly with it which would yield better performance than training it by itself.

An aim of this experiment was to determine which dataset had the most positive interaction with a particular dataset. Table 2 shows the result of this in the ‘Best Dataset’ column. Most of the datasets which proved to be the best combined with a given dataset were predictable in that datasets which annotated the same named entities were able to help each other, but other successful combinations were less predictable, for example the dataset which best interacted with BC4CHEMD (Chemical) was BioNLP13GE (Gene/Protein) despite the presence of other datasets which annotated Chemicals and the dataset which best interacted with Linnaeus (Species) was NCBI-Disease (Disease) not another dataset which annotated Species.

The full list of results from the 15 × 14 models were not included here for brevity, but they can be found in section 2 of Additional file 1.

### Multi-task learning in grouped datasets

The results in Tables 3, 4, 5, 6, 7 and 8 present the effect of training the Multi-output model with datasets which aim to annotate similar named entities. In four of the six groups, there were marked increases in the average performance of the group of tasks, marked decrease in one group and the results of the remaining one were equivalent. Across the groups there were 27 experiments; 16 showed significant increase, 1 showed significant decrease and the remaining 10 showed no significant change.

**Table 3:** Chemical group

Dataset	STM	MO-MTM
BC4CHEMD	**82.95**	82.51
BC5CDR	87.02	89.22*
BioNLP11ID	**65.79**	63.74
BioNLP13CG	66.40	77.17*
BioNLP13PC	74.53	79.46*
CRAFT	**80.00**	74.83
Average	76.43	77.49

(**Bold**: best scores, *: statistically significant)

**Table 4:** Species group

Dataset	STM	MO-MTM
BioNLP11ID	74.14	77.25*
BioNLP13CG	82.75	86.29*
CRAFT	**97.74**	97.44
Linnaeus	**83.98**	83.54
Average	84.65	86.13

(**Bold**: best scores, *: statistically significant)

**Table 5:** Cellular component group

Dataset	STM	MO-MTM
BioNLP13CG	72.79	74.80*
BioNLP13PC	83.23	84.67*
CRAFT	61.04	63.08*
Average	72.35	74.18

(**Bold**: best scores, *: statistically significant)

**Table 6:** Disease group

Dataset	STM	MO-MTM
BC5CDR	**80.46**	80.39
NCBI-Disease	80.26	**80.46**
Average	80.36	80.42

(**Bold**: best scores, *: statistically significant)

**Table 7:** Cell group

Dataset	STM	MO-MTM
BioNLP13CG	**83.25**	82.83
CRAFT	**88.08**	86.89*
Average	85.66	84.86

(**Bold**: best scores, *: statistically significant)

**Table 8:** Gene/protein group

Dataset	STM	MO-MTM
BC2GM	72.63	**73.04**
BioNLP09	83.90	84.76*
BioNLP11EPI	77.72	79.00*
BioNLP11ID	86.20	87.21*
BioNLP13CG	83.40	85.98*
BioNLP13GE	73.28	79.66*
BioNLP13PC	83.21	84.84*
CRAFT	72.85	75.16*
Ex-PTM	68.56	74.91*
JNLPBA	69.60	**69.73**
Average	77.14	79.43

(**Bold**: best scores, *: statistically significant)

It is important to note that although the focus of the annotations were similar, both the sources of the text and the annotations are different for these datasets. This general improvement suggests that the multi-task model was able to utilize the real-world distributions from which these labeled examples were sampled and leverage information in all or some of them to increase performance in most of them, despite variations in source text and possibly annotation guidelines. This provides evidence of MTL having a positive effect on the NER task.

### Multi-task learning on all datasets

The results in Table 9 show the effect of training the Multi-output multi-task model and the Dependent multi-task model with all the datasets as they were originally annotated. These results show that the average score of the Multi-output model is higher than that of the 15 separately trained models. Since the average score over such varied datasets as those used can be misleading, we examined each dataset individually and analyzed the differences in performance.

**Table 9:** Single task and multi-task f-scores on NER tasks

Dataset	Baseline	STM	MO-MTM	D-MTM
AnatEM	81.79	81.55	81.83	82.21*
BC2GM	70.31	72.63	**73.17**	72.87
BC4CHEMD	81.08	82.95	82.37	**83.02**
BC5CDR	83.11	83.66	**83.90**	83.83
BioNLP09	81.84	83.90	**84.20**	84.10
BioNLP11EPI	74.98	77.72	78.86*	78.03*
BioNLP11ID	81.44	81.50	80.58*	**81.73**
BioNLP13CG	75.23	76.74	78.90*	77.52*
BioNLP13GE	72.49	73.28	78.58*	74.00*
BioNLP13PC	79.35	80.61	81.92*	81.50*
CRAFT	78.76	79.55	79.10	**79.56**
Ex-PTM	65.75	68.56	74.90*	69.67*
JNLPBA	67.43	69.60	**70.09**	69.44
Linnaeus	79.01	83.98	81.57	**84.04**
NCBI-Disease	79.09	80.26	79.02	**80.37**
Average	76.78	78.43	79.26	78.79

(**Bold**: best scores, *: statistically significant compared to single-task model)

This revealed that of the results for individual datasets, there were 6 where the difference in performance between the Multi-output model and the single-task model was statistically significant. There were 5 datasets where it performed significantly better and 1 dataset where it was significantly worse. The performances in the 9 remaining datasets were comparable. This also provides evidence of MTL having a positive effect on the NER task as in the “Multi-task learning in grouped datasets” section but in this case it is a more impressive feat since the number of datasets and the variability among them are much increased.

Table 9 also illustrates that the average score of the Dependent model was higher than that of the 15 separately trained models. Analysis of the results revealed that of the results for individual datasets, there were 6 where the difference in performance between that and the single-task model was significant. In all 6 it performed significantly better, it was significantly worse in none and the performances in the 9 remaining datasets were comparable.

These results show the advantages and disadvantages of the two approaches to MTL which each model incorporates. In the Dependent model the average improvement was less impressive than the Multi-output model but it also shows that this model did not make performance on any particular dataset significantly worse. This is possibly due to the large amount of separation between the components responsible for each task which allows for the NER model to incorporate POS information when it can be helpful and ignore it when it is not. Comparison of the results of the Multi-output model and the Dependent Model show that the Multi-output model had a higher average score because it gave larger gains in the datasets where it performed better but also showed larger losses where it did not. This is possibly due to sharing most of the model among the datasets regardless of whether or not this is helpful. This result indicates that in cases where tasks are thought to be similar and can contribute equally the Multi-output model may be the better of the two while in cases where there is a clear main and auxiliary task separation, the Dependent model may perform better.

There were seven datasets which showed significant performance change across the two multi-task models. Five of them (BioNLP11EPI, BioNLP13CG, BioNLP13GE, BioNLP13PC, Ex-PTM) were improved in both models which indicated that these datasets benefited from simply having the information present in the additional datasets available to them, regardless of the model. One (AnatEM) had better performance in the Dependent model but no difference in the Multi-output model while another (BioNLP11ID) had significantly worse performance in the Multi-output model but no significant performance change in the Dependent model. Both of these datasets recorded improved performance in the Dependent model which indicate that they benefit from having POS-Tagging information integrated in the manner which the Dependent model uses.

### Dataset size and multi-task learning

Table 10 correlates dataset performance and decreased size both in isolation and when trained in a multi-task setting. The best scores for each dataset is in bold and the better scores for each training set size are italicized. Statistically significant changes in scores relative to the full single-task model are shown with asterisks while statistically significant changes in scores relative to the corresponding single-task model are marked with a plus sign.

**Table 10:** Effect of dataset size reduction on single-task and multi-task performance

	1.0	0.5	0.5	0.25	0.25	0.1	0.1
Dataset	STM	STM	MO-MTM	STM	MO-MTM	STM	MO-MTM
AnatEM	**81.55**	78.74*	78.35*	74.82*	76.59*+	65.99*	63.15
BC2GM	**72.63**	70.27*	70.73*+	67.37*	67.14*	63.07*	63.14*
BC4CHEMD	**82.95**	80.16*	79.22*+	76.81*	76.26*	71.94*	72.53*
BC5CDR	**83.66**	81.15*	82.45*+	79.09*	80.44*+	74.47*	75.48*
BioNLP09	**83.90**	81.89*	82.22*	80.56*	79.58*	75.12*	78.32*
BioNLP11EPI	**77.72**	74.00*	77.57*+	70.89*	*75.61+*	67.63*	75.04*+
BioNLP11ID	**81.50**	76.65	*81.39*	70.60*	78.17*+	68.19*	73.52*
BioNLP13CG	**76.74**	70.58*	75.02*+	65.08*	72.98*+	51.61*	67.86*+
BioNLP13GE	73.28	73.32	81.37*+	67.43	78.80*	52.66*	77.12*+
BioNLP13PC	**80.61**	75.39*	*77.57*	70.03*	73.90*	57.62*	68.65*+
CRAFT	**79.55**	75.25*	*79.01+*	72.19*	76.79*+	60.91*	71.00*
Ex-PTM	68.56	62.81	74.60*+	53.30*	74.27*+	47.01*	*69.83+*
JNLPBA	69.60	68.34	***69.65***	66.63*	*68.13*	62.80*	65.40*+
Linnaeus	83.98	80.08*	***87.61+***	69.53*	*79.86*	39.44	*45.73*
NCBI-Disease	**80.26**	76.51	*76.84*	71.88*	73.55*	67.48*	62.89*
Average	78.43	75.01	78.24	70.41	75.47	61.73	68.64

(**Bold**: best scores for dataset, *Italic:* better score for each setting, *: statistically significant compared to full single-task model, +: statistically significant compared to corresponding single-task model)

Multi-task Learning is advantageous here as well as shown in the ‘0.5 MO-MTM’, ‘0.25 MO-MTM’ and ‘0.1 MO-MTM’ columns. As the size of the datasets were reduced, the multi-task model was able to show an increase in average score over the corresponding single-task models. The gap between the average scores of the single-task models and the corresponding multi-task model also widened as the datasets became smaller. In fact, there were two datasets (BioNLP13GE and Ex-PTM) where using only 50% of the training data in a multi-task setting yielded significantly better performance than using the full training data in a single task setting. In the case of Ex-PTM, this was also the case when it was used with only 25% of its training data. This augurs well for our stated aim of using Multi-task Learning to improve performance on small datasets. It can also indicate that new datasets can contain fewer annotations and thus would consume less resources to create - another stated aim of this work.

An additional result from this experiment was that, for many of the datasets, randomly removing 50% of the training data resulted in an average drop of only approximately 3.4% F-score in single task training as can be seen by comparing the ‘1.0 STM’ and ‘0.5 STM’ columns of Table 10. When the model is trained on 75% less training data, that average drop extends to 8% as some datasets continue to be robust although there is a predictable drop in performance in most datasets. It is not until 90% of the training data of the datasets are removed that a steep drop in average performance of approximately 16.7% is registered across all datasets. This high performance on reduced-sized corpora supports what is reported in [36] using BANNER [37], a NER model based on Conditional Random Fields (CRF) for biomedical NER. This may indicate that, like BANNER, the single-task model presented in the “Single task model” section is able to efficiently utilize even a relatively small amount of training data to obtain good enough performance. We wish to point out that in the respective data reduction scenarios, the multi-task models record drops of approximately 0.2% when 50% of the training data is removed, approximately 3.0% when 75% is removed and approximately 9.8% when 90% is removed.

### Comparison with benchmark results

The focus of this study is on Multi-task Learning and we have chosen not to perform task-specific adaptation or use resources such as gazetteers that are frequently part of state-of-the-art methods targeting individual corpora or particular entity types. It is nevertheless an interesting question how the level of performance achieved by our methods compares to that of competitive task-specific systems. To address this question, we surveyed the literature on each of the corpora to identify results representative of the best-performing methods for each. In particular, for the corpora introduced for shared tasks involving named entity recognition (BC2GM, BC4CHEMD, BC5CDR, and JNLPBA) we consider the highest result reported in the shared task for our benchmark.

To assure that our results are comparable to previously published ones, we apply the same evaluation metrics and criteria as in each of the studies compared to. When those criteria differ from the exact mention-level F-score used in our primary evaluation, we further apply the specific software released for evaluation using each corpus to assess performance, i.e. the evalbio.pl script for AnatEM and alt_eval.perl for BC2GM. For the other corpora, we use the standard conlleval.pl evaluation introduced for CoNLL shared tasks.

The BioNLP corpora (BioNLP09, BioNLP11EPI, BioNLP11ID, BioNLP13CG, BioNLP13GE and BioNLP13PC) and the Ex-PTM corpus were introduced for event extraction tasks where gold named entity annotations are taken to be available as a starting point for the task. Thus, although the annotations of these corpora can be readily used for NER as we have done here, there is no previous body of NER work establishing state-of-the-art performance on these resources. Similarly, the CRAFT corpus was not primarily designed for NER and has not been previously used for sequential labeling tasks of the form we consider here. For these reasons, in the following comparison we focus on the remaining corpora: AnatEM, BC2GM, BC4CHEMD, BC5CDR, JNLPBA, Linnaeus, and NCBI-Disease.

#### AnatEM

The AnatEM corpus was created for anatomical entity mention recognition and released with a benchmark system, AnatomyTagger, which scored 91.61% F-score for right boundary match in the single-class setting we apply in this study [38]. To the best of our knowledge this result remains the state of the art for this corpus.

#### BC2GM

The top-performing system [39] in the BioCreative II gene mention recognition task achieved an F-score of 87.21% by the official task evaluation criteria, which relax strict entity span matching by defining alternative boundaries for some named entities [2]. We note that this result has remained very competitive, with recent systems reporting similar results (see e.g. [40]).

#### BC4CHEMD

In the original BioCreative IV chemical entity mention recognition task [3], the highest performance, 87.39% F-score, was achieved by the tmChem system of Leaman et al. [41]. The task required exact matching of gold entities, i.e. the same criterion applied in our primary evaluation.

#### BC5CDR

The recent BioCreative V Chemical Disease Relation task [5] included an evaluation of the mention-level performance of chemical and disease mention recognition, the subtask we consider in this paper. The best-performing system for this task, by Li et al. [42], achieved an F-score of 86.76% under standard exact matching criteria.

#### JNLPBA

The highest performance in the 2004 JNLPBA shared task on biomedical entity recognition was achieved by the system of Zhou and Su [43], which scored 72.55% F-score for exact match [44]. Although this result is notably older than many of the other benchmarks considered here, it remains competitive with the performance of recently proposed approaches (e.g. [45]).

#### Linnaeus

As for AnatEM, the Linnaeus corpus was created specifically for entity mention (specifically, species name) recognition and released together with a recognition system. The original study reports the performance of the system as 94.3% recall and 97.1% precision (95.68% F-score) on the mention level [4]. A number of caveats to comparability apply to the evaluation on on this corpus. First, as the Linnaeus system is dictionary-based and thus requires no training data, it was evaluated on the entire corpus rather than on a specific test subset (as we do here). Second, later work by [46] reported a notably lower F-score of 85.1% for the Linnaeus system on this corpus in an evaluation where their proposed tagger, SPECIES, achieved 91.1%. While comparability to our results may thus be lower than for the other corpora, we nevertheless reference the highest number, reported by Gerner et al. [4] as our benchmark here.

#### NCBI-Disease

The NCBI disease corpus was introduced for disease name recognition and normalization and has been applied in numerous studies of this task [6]. For this corpus, we select as our benchmark a result from the TaggerOne system recently introduced by two of the authors of the corpus [47]. TaggerOne achieved an exact match F-score of 82.9%, a result that is highly competitive with other recent work on the corpus (e.g. [48]).

Table 11 shows the benchmark results and the results achieved by the methods considered here when the same evaluation criteria are applied.

**Table 11:** Comparison to benchmark results

Corpus	Benchmark	Ours	Matching criteria
AnatEM	91.61	88.55	Right boundary match
BC2GM	87.21	84.41	Alternative boundaries
BC4CHEMD	87.39	82.32	Exact
BC5CDR	86.76	83.87	Exact
JNLPBA	72.55	68.95	Exact
Linnaeus	95.68*	79.33	Exact
NCBI-Disease	82.9	77.82	Exact

(*: see text for caveat regarding comparability)

### Applications and practicality

The argument can be made that the increases in performance we report are trivial and may not be worth doing in practical applications. This can be especially true of the Dependent multi-task model. We note however that, if there is no benefit from Multi-task Learning, then the single-task setting can be used for a particular task and the practitioner is no worse off than before. Our contribution is that for some datasets the benefits can be significant and in those cases we present an option to the practitioner to obtain improved performance which previously was not available. An additional argument against application of the work presented is the results which show that it can be difficult to predict when Multi-task Learning will be beneficial and by how much. We contend that the models and methods presented here make it possible to quickly determine empirically the amount of benefit that Multi-task Learning, as implemented here, provides.

The training time of the models varied according to the size of the dataset(s) involved and the type of model. The experiment which took the longest time to run was the one where all the datasets were trained together with the Multi-output multi-task model which we ran for 190,000 steps with batch sizes of 200 examples drawn on each step from a randomly selected dataset. This took approximately 40 min to train on a single Nvidia Titan X GPU. As the weights are randomly initialized at the start of training, there is some variation in scores between runs. For the single task experiments, the average variance in F-Score was 0.099. For the Multi-output multi-task model it was 0.092 and for the Dependent multi-task model it was 0.012. In our experiments under the conditions outlined here, training never failed entirely.

## Conclusion

In this paper we investigated whether Multi-task Learning could benefit the key text mining task of biomedical NER across various NER datasets. We first developed a single task CNN model for NER and then two variants of a multi-task CNN. We trained these on 15 domain-specific datasets representing a myriad of biomedical named entities.

We observed an average improvement on Multi-task Learning in comparison with single task learning. Individually, there were also significant improvements on many of the datasets. Although there was a drop in performance on some tasks, for most tasks performance improves significantly. This is a promising result which shows the potential of MTL for biomedical NER.

We have made all the datasets used and the code of all our models publicly available for download and use along with instructions of how the models can be trained using the data. These can be found at https://github.com/cambridgeltl/MTL-Bioinformatics-2016. We presented our experiments along with the datasets and models which demonstrated improvement and detailed the conditions under which they did so.

Limitations to the work include that it can be difficult to predict situations when these Multi-task Learning models will definitely provide benefit and the extent of any increases in performance that they may give before it is actually applied. This area has recently received research attention [49–51] and some of the proposed methods may be useful in this regard in the future. Another limitation is that the current implementation of the models does not allow for overlapping annotations of the same term in the data.

### Future work

The field of biomedical NLP contain several challenging tasks, many of a more complex nature than NER and POS-tagging but which use those tasks as starting points. There are also freely available datasets for some of these tasks. Our presented models and methods are flexible enough to apply to some of these tasks (e.g. event extraction) and it would be interesting to see if the results presented here can also be produced on some of them.

Complex tasks usually utilise information from less complex tasks (e.g. core event detection utilising NER). Using the Dependent multi-task model presented here, which experimented with an architecture that facilitates utilizing lower-level tasks to aid higher-level ones in a single network, is a plausible approach to handling these tasks in light of the promising results presented here.

## Additional file

Additional file 1 This document contains supplementary information for the paper: A Neural Network MultiTask Learning Approach to Biomedical Named Entity Recognition. (PDF 176 kb)

## Abbreviations

CNN: Convolutional neural network
MTL: Multi-task learning
NER: Named entity recognition
NLP: Natural language processing
POS-Tagging: Part of speech tagging
ReLU: Rectified linear unit
