# Supplementary material for: A neural network multi-task learning approach to biomedical named entity recognition
Source: BMC Bioinformatics. 2017 Aug 15;18:368. doi: 10.1186/s12859-017-1776-8 (PMC5558737; doi:10.1186/s12859-017-1776-8)
Supplement: Additional file 1 — This document contains supplementary information for the paper: A Neural Network MultiTask Learning Approach to Biomedical Named Entity Recognition. (PDF 176 kb) [file 12859_2017_1776_MOESM1_ESM.pdf]

Supplementary Information: A Neural Network Multi-Task Learning Approach to Biomedical Named Entity Recognition

Gamal Crichton, Sampo Pyysalo, Billy Chiu, Anna Korhonen

This document contains supplementary information for the paper *A Neural Network Multi-Task Learning Approach to Biomedical Named Entity Recognition.*

1 Datasets

We used 16 biomedical corpora representing 15 NER corpora and one part-of-speech (POS) corpus. Details of their creation, prior use, and conversion into the CoNLL format used to train, develop and test our methods are presented in the following.

1.1 AnatEM corpus

The extended Anatomical Entity Mention corpus (Pyysalo and Ananiadou, 2013) is the result of combining and extending the Anatomical Entity Mention (AnEM) corpus (Ohta et al., 2012) and the Multi-level Event Extraction corpus (MLEE) (Pyysalo et al., 2012a). AnEM consists of 500 randomly selected PubMed abstracts and full-text extracts annotated for anatomical entity mentions. MLEE consists of 262 PubMed abstracts on the molecular mechanisms of cancer, specifically relating to angiogenesis. MLEE is also annotated for anatomical entities specified in AnEM.

AnatEM was created by combining the anatomical entity annotations of the AnEM and MLEE corpora, then manual annotation was done on an additional 100 documents following the selection criteria of AnEM and 350 documents following those of MLEE, for a selection of topics related to cancer. The resulting corpus thus consists of 1212 documents, 600 of which are drawn randomly from abstracts and full texts as in AnEM, and the remaining 612 are a targeted selection of PubMed abstracts relating to the molecular mechanisms of cancer.

Conversion The AnatEM corpus data is available from `http://nactem.ac.uk/anatomytagger/` in multiple formats, including CoNLL-style IOB, and is provided with a pre-defined split into train, development and test subsets. We use this data in a single-class NER setting, mapping all NE labels to ANATOMY, but otherwise without modification; the number of annotations and their spans are thus identical to the source data.

1.2 BC2GM corpus

The BioCreative II Gene Mention (BC2GM) task corpus consists of 20,000 sentences from biomedical publication abstracts and is annotated for mentions of the names of genes, proteins and related entities using the single NE class GENE (Smith et al., 2008). It has become the major NER benchmark for gene/proteins names and has been used to train and evaluate many available NER systems such as BANNER (Leaman and Gonzalez, 2008) and Gimli (Campos et al., 2013).

Conversion The BC2GM corpus is available from `http://www.biocreative.org/` in a custom standoff format and a standard train/test split. We created a development set by splitting off 2,500 sentences from the training data and converted the corpus into CoNLL format using tools available from `https://github.com/spyysalo/bc2gm-corpus`.

The BC2GM corpus has the unique feature of defining alternative boundaries for some of the annotated names. For the conversion, we only used the primary annotations (`GENE.eval` files), which could be represented highly accurately in the CoNLL format: the converted data contained 99.95% of the number of annotations in the original. No differences from token boundaries were introduced: all names in the converted data matched names in the source data.

1.3 BC4CHEMD corpus

The BioCreative IV Chemical and Drug (BC4CHEMD) named entity recognition task corpus consists of 10,000 abstracts annotated for mentions of chemical and drug names using a single class, Chemical (Krallinger et al., 2015).

Conversion The BC4CHEMD corpus data is available from `http://www.biocreative.org/` in a TAB-separated standoff format and defines standard training, development and test subsets. We converted the data into CoNLL format using custom tools available from `https://github.com/spyysalo/chemdner-corpora`, mapping non-ASCII characters to ASCII. The basic conversion is highly accurate; the number of annotations in the converted data is 99.95% of that in the source. Non-ASCII characters in the source and tokenization differences lowered the number of matching strings somewhat, to 97.16%.

1.4 BC5CDR corpus

The BioCreative V Chemical Disease Relation (CDR) corpus was created for the BioCreative V Chemical Disease Relation (CDR) Task (Wei et al., 2015) and consists of human annotations of all chemicals, diseases and their interactions in 1,500 PubMed articles. 1,400 of these articles were selected from an existing 150,000 chemical-disease interactions which were annotated by CTD-Pfizer. The CTD biocurators followed CTDs rigorous curation process and curated interactions from mostly just the abstract, but referenced the full text when it was necessary to resolve relevant issues mentioned in the abstract. The remaining 100 articles were completely new.

Conversion The BC5CDR corpus is available in BioC (Comeau et al., 2013) and PubTator (Wei et al., 2013) formats from `http://www.biocreative.org/` with pre-defined training, development and test subsets. We converted the chemical and disease annotations of the corpus from the PubTator format using tools available from `https://github.com/spyysalo/pubtator`. The conversion introduced only minimal divergence, increasing the annotation number by two to 100.01% of the original due to sentence splitting errors inside annotation spans. 99.94% of the annotated strings in the source match those in the converted data, reflecting rare instances where annotation boundaries occurred inside alphanumeric tokens.

1.5 BioNLP09 corpus

The BioNLP'09 shared task on event extraction (Kim et al., 2009) targeted semantically rich event extraction, involving the extraction of several different classes of information. To focus on these novel aspects of the event extraction task, it was assumed that NER has already been performed and the task began with a given set of gold protein annotations. The named entities in the BioNLP task data were prepared based on the GENIA event corpus. Part of the data were derived from the publicly available event corpus (Kim et al., 2008), and the remainder from an unpublished portion of the corpus.

Conversion The BioNLP'09 shared task data is available from `www.nactem.ac.uk/tsujii/GENIA/SharedTask/` in the `.ann` standoff format first introduced for the task. We use the Protein annotations of the corpus (the only physical entity annotations released also for its test data) and the training, development and test split of the original dataset. The data was converted from standoff to the CoNLL format using the `standoff2conll` tool available from `https://github.com/spyysalo/standoff2conll`.

After conversion, the number of annotations was 99.96% of the number in the source, and 99.69% of names in the original data matched names in the converted data (ignoring whitespace), indicating that almost all of the original annotations could be exactly represented in the CoNLL format with the applied tokenization.

1.6 BioNLP11 corpora

Similar to the BioNLP'09 task, the BioNLP Shared Task 2011 (Kim et al., 2011; Pyysalo et al., 2012b) was focused on semantically rich tasks such as Infectious Diseases (ID) and Epigenetics and Post-translational Modifications (EPI). The ID task was concerned with the molecular mechanisms of infection, virulence and resistance while the EPI task focused on the extraction of statements regarding chemical modifications of DNA and proteins. Both tasks used manual annotations created specifically for the shared task, with automatic support for the initial tagging of named entities.

The texts for the EPI task corpus were drawn from PubMed abstracts annotated with the MeSH term corresponding to the target event (e.g. Acetylation). Protein/Gene entity mentions in the selected abstracts were automatically tagged using the BANNER (Leaman and Gonzalez, 2008) named entity tagger trained on the GENETAG (Tanabe et al., 2005) corpus. Abstracts where fewer than five entities are found were removed and documents not relevant to the targeted topic were also manually removed.

The data for the ID corpus were drawn from the primary text content of full-text PMC open access documents deemed by infectious diseases domain experts to be representative publications on two-component regulatory systems. The annotation of the Protein entities was performed automatically using NeMine (Sasaki et al., 2008) trained on the JNLPBA data (Kim et al., 2004) with threshold 0.05, filtered to only GENE and Protein types.

Conversion The BioNLP'11 corpora are available from `http://2011.bionlp-st.org/` in the standoff format used for the BioNLP'09 data (Section 1.5). We use the standard training, development and test sets of each of the BioNLP'11 corpora and all physical entity annotations released for all subsets of the two corpora. Conversion was performed with the `standoff2conll` tool. As the BioNLP'11 ID task data contained a large number of annotations where more than one name occurred inside the span of another annotation (e.g. REGULON-OPERON or TWO-COMPONENT-SYSTEM), we resolved overlaps in favor of keeping the shorter of any pair of overlapping annotations,[1] thus maximizing the number of annotations carried over from the source. Notably, this overlap pattern occurred for all 492 TWO-COMPONENT-SYSTEM annotations in the corpus (3.8% of all annotations), leading to the elimination of this annotation type from the converted data.

The converted EPI data contains 99.87% of the number of annotations in the source, but just 94.86% of originals matched converted in text, reflecting a comparatively high number of cases where an annotation boundary occurred within an alphanumeric token. For ID, the number of annotations fell to 86.99% in conversion, reflecting the frequent pattern of annotation overlap. The fraction of matching names was 85.53%, indicating that annotation boundaries rarely differ from token boundaries.

1.7 BioNLP13 corpora

The BioNLP 2013 Shared Task focused on knowledge-based construction. There were six tasks in this Shared Task, of which three datasets were used for our work: GENIA Event Extraction (GE), Cancer Genetics (CG) and Pathway Curation (PC).

The GE corpus consists of 20 full paper articles sourced from PubMed Central Open Access subset (PMCOA) with 7721 spans manually annotated as protein names (Kim et al., 2013). The CG task corpus consists of 600 PubMed abstracts annotated for over 17,000 events and was prepared as an extension of the MLEE (Pyysalo et al., 2012a) corpus of 250 abstracts (c.f.

[1]Option `-o keep-shorter` for `standoff2conll`

Section 1.1). The PC task corpus consists of 525 PubMed abstracts, chosen for the relevance to specific pathway reactions selected from SBML models registered in BioModels and PANTHER DB repositories (Mi and Thomas, 2009). The corpus was manually annotated for over 12,000 events on top of close to 16,000 entities.

Conversion The BioNLP'13 corpora are available from `http://2013.bionlp-st.org/` in the same standoff format as the '09 and '11 corpora (Sections 1.5 and 1.6). As for these resources, we use the standard training, development and test set splits of each corpus and all of the physical entity annotations available for each dataset, and perform the conversion using the `standoff2conll` tool. Of the BioNLP'13 corpora only the CG task involved overlap between annotations in the source data; these were resolved in favor of keeping the shorter annotations, as for BioNLP'11 ID processing.

The conversion was highly accurate for all three of the BioNLP'13 corpora: the numbers of annotations in the converted data were 99.07%, 99.91%, and 99.95% of the numbers of annotations in the source for CG, GE, and PC respectively. Similarly, the fractions of annotated strings matching after conversion were 98.67%, 98.79%, and 99.80% (resp.).

1.8 Colorado Richly Annotated Full Text (CRAFT) corpus

The CRAFT corpus (Bada et al., 2012; Verspoor et al., 2012) consists of 67 full-text articles, over 790,000 Tokens, over 21,000 Sentences and approximately 140,000 concept annotations. It manually annotates all mentions of nearly all concepts from nine prominent biomedical ontologies and terminologies: Cell Type Ontology, Chemical Entities of Biological Interest ontology, NCBI Taxonomy, Protein Ontology, Sequence Ontology, Entrez Gene database entries, and the three sub-ontologies of the Gene Ontology. There was emphasis on journal articles that comprise the corpus being drawn from diverse biomedical disciplines and on them being completely annotated. We use the annotated physical entities from this corpus.

1.8.1 Conversion

The 67 publicly released articles of the CRAFT corpus are available in multiple formats from `http://bionlp-corpora.sourceforge.net/CRAFT/`. We split the data into 34 training, 11 development and 22 test documents and created a custom conversion for the corpus from the Knowtator format (Ogren, 2006). Of the resources considered in this study, the CRAFT term annotations represented the most challenges for use in sequence labeling: these are frequently overlapping, occasionally discontinuous, and associated with ontology identifiers (e.g. PR:000009758) rather than simple labels such as PROTEIN. To convert the corpus, we first excluded annotations not associated with physical entity types (biological process/molecular function, coreference, sections and typography). We then merged annotations associated with gene (ENTREZGENE) and protein (PR) identifiers, which frequently mark identical spans in the source data, into a single gene/gene-product type. We likewise merged those referencing ORGANISM and TAXONOMIC RANK vocabularies. We finally deduplicated the resulting annotations and resolved remaining overlapping and discontinuous entities with corpus-specific heuristics implemented in a custom tool available from `https://github.com/spyysalo/knowtator2standoff/`.

The resulting dataset contains 72.05% of the number of annotations in the physical entity-associated subsets of CRAFT (CHEBI, CL, ENTREZGENE, GO-CC, NCBITAXON, PR, and SO), with 69.76% of the annotated names in the source matching ones in the converted data. These numbers are by far the lowest among the corpora considered here. While most of the difference reflects fundamental limitations of the BIO representation, many decisions in the conversion could reasonably be made in another way and our results on CRAFT should thus not be directly compared to others where a different conversion of the data has been used.

1.9 Ex-PTM corpus

The Exhaustive Post-Translational Modifications corpus (Pyysalo et al., 2011) was part of the BioNLP Shared Task 2011 and employed a similar creation methodology to that of the BioNLP11

EPI task corpus (c.f. Subsection 1.6). It annotated 360 PubMed abstracts containing 76,806 words of which 4,698 were annotated as proteins. Though the more semantically complex PTM identification task used manual annotations, the Protein/Gene entity mentions were automatically tagged using the BANNER (Leaman and Gonzalez, 2008) named entity tagger trained on the GENETAG (Tanabe et al., 2005) corpus. Abstracts containing fewer than five entities were removed and a randomly chosen subset of the remaining documents were annotated.

Conversion The Exhaustive PTM corpus is available from `http://www.geniaproject.org/` in the standoff format used by the BioNLP corpora (Section 1.5). Unlike the shared task resources, the Ex-PTM corpus does not come with a pre-defined development set, but only a split between training and test data; we thus split off 49 of the 196 test documents as a development set. Conversion of the single physical entity annotation type, PROTEIN, was again performed with `standoff2conll`. As the source data contained a small number of non-ASCII characters, the conversion tool was run with the `-a` option to map these to ASCII.

The conversion exactly preserves the number of annotations in the source data. However, as for the BioNLP'11 EPI corpus (Section 1.6) with which the Ex-PTM corpus shares a domain and some development history, the fraction of original names matching the text of converted names is notably lower at 95.72%, reflecting comparatively frequent entity mention boundaries inside alphanumeric tokens.

1.10 JNLPBA corpus

The Joint workshop on NLP in Biomedicine and its Applications corpus consists of 2,404 publication abstracts (approx. 22,400 sentences) and is annotated for mentions of five entity types: CELL LINE, CELL TYPE, DNA, RNA, and PROTEIN (Kim et al., 2004). The corpus was derived from GENIA corpus entity annotations. It is now a standard point of reference for evaluating multi-class biomedical entity taggers and has served as training material for tools such as ABNER (Settles, 2005) and the GENIA Tagger.

Conversion The JNLPBA corpus is available from `http://www.geniaproject.org/` and distributed in the CoNLL IOB format with a split into train and test subsets. To create the development set, we separated 200 of the 2000 documents from the training data. As format conversion was not required, the annotations match the original data exactly.

1.11 LINNAEUS corpus

The LINNAEUS corpus (Gerner et al., 2010) consists of 100 full-text documents from the PMCOA document set which were randomly selected. All mentions of species terms were manually annotated and normalized to the NCBI taxonomy IDs of the intended species.

Conversion The LINNAEUS corpus is available from `http://linnaeus.sourceforge.net/` in a TAB-separated standoff format. The resource does not define training, development or test subsets. We converted the corpus into BioNLP shared task standoff format using a custom script available from `https://github.com/spyysalo/linnaeus-corpus`, split it into 50-, 17-, and 33-document training, development and test sets, and then converted these into the CoNLL format using `standoff2conll`. As a full-text corpus, LINNAEUS contains comparatively frequent non-ASCII characters, which were mapped to ASCII using the `standoff2conll -a` option.

The conversion was highly accurate, but due to sentence-splitting errors within entity mentions, the number of annotations in the converted data was larger by four (100.09%) than that in the source data. 99.77% of names in the original annotation matched names in the converted data.

1.12 NCBI Disease corpus

The NCBI Disease corpus (Doğan et al., 2014) consists of 793 PubMed abstracts fully annotated at the mention and concept level for disease mentions. The public release of the NCBI disease

corpus contains 6,892 disease mentions, which are mapped to 790 unique disease concepts. Of these, 88% link to a MeSH identifier, while the rest contain an OMIM identifier. 91% of the mentions were linked to a single disease concept, while the rest are described as a combination of concepts.

Conversion The NCBI Disease corpus is available in a TAB-separated standoff format with a standard split into training, development and test subsets from `http://www.ncbi.nlm.nih.gov/CBBresearch/Dogan/DISEASE/`. We converted the corpus annotations to CoNLL format using tools available from `https://github.com/spyysalo/ncbi-disease`. The converted number of annotations was 99.84% of the original number, with 99.81% of strings in the original annotations matching with converted data. The differences were mostly due to a duplicated document in the source data.

1.13 GENIA POS

The GENIA corpus is one of the most widely used resources for biomedical NLP and has a rich set of annotations including parts of speech, phrase structure syntax, entity mentions, and events (Ohta et al., 2002). For this work we use the GENIA POS annotations, which cover 2000 PubMed abstracts (approx. 20,000 sentences).

Conversion We use the GENIA corpus v3.02 POS annotations that were used to train the GENIA tagger (Tsuruoka et al., 2005), available from `https://github.com/spyysalo/genia-pos`.[2] We split off 210 of the 1790 training set documents into a development test. The data is distributed in a tagged-token format that could be straightforwardly recast into the CoNLL format, preserving both the tokenization and the annotations of the original exactly.

2 Full Effects Results

To determine the exact effect that each NER dataset had on every other one, the multi-task model described in the paper was used to train each NER dataset with every other one. That is, a Multi-output multi-task model was trained for each ordered combination of the datasets to give 15 x 14 models. The best results for each dataset was included in the paper, but the full set of all results could not be included for space considerations. They are added in Table 1.

Table 1: Full Effects Results. (*: best score)

Dataset	Scores
AnatEM	**BC2GM**: 80.63, **BC4CHEMD**: 77.72, **BC5CDR**: 80.85, **BioNLP09**: 80.99, **BioNLP11EPI**: 80.81, **BioNLP11ID**: 81.22, **BioNLP13CG**: 81.14, **BioNLP13GE**: 81.48, **BioNLP13PC**: 81.03, **CRAFT**: 80.03, **Ex-PTM**: 81.57, **JNLPBA**: 78.20, **Linnaeus**: 80.94, **NCBI-Disease**: 81.68*
BC2GM	**AnatEM**: 72.07, **BC4CHEMD**: 68.32, **BC5CDR**: 71.80, **BioNLP09**: 71.43, **BioNLP11EPI**: 71.95, **BioNLP11ID**: 71.56, **BioNLP13CG**: 71.68, **BioNLP13GE**: 72.17, **BioNLP13PC**: 72.04, **CRAFT**: 70.20, **Ex-PTM**: 72.21*, **JNLPBA**: 69.35, **Linnaeus**: 71.64, **NCBI-Disease**: 71.84
BC4CHEMD	**AnatEM**: 79.58, **BC2GM**: 78.84, **BC5CDR**: 79.43, **BioNLP09**: 79.34 **BioNLP11EPI**: 79.91, **BioNLP11ID**: 79.35, **BioNLP13CG**: 78.98, **BioNLP13GE**: 80.31*, **BioNLP13PC**: 79.54, **CRAFT**: 78.19, **Ex-PTM**: 80.29, **JNLPBA**: 77.37, **Linnaeus**: 79.39, **NCBI-Disease**: 79.57
BC5CDR	**AnatEM**: 83.21, **BC2GM**: 82.54, **BC4CHEMD**: 81.45, **BioNLP09**: 83.18, **BioNLP11EPI**: 83.77*, **BioNLP11ID**: 83.38, **BioNLP13CG**: 83.66, **BioNLP13GE**: 83.54, **BioNLP13PC**: 83.58, **CRAFT**: 81.95, **Ex-PTM**: 83.03,

[2]We are grateful to Yoshimasa Tsuruoka for providing this version of the corpus, which differs from that available from `http://www.geniaproject.org/` most importantly in providing a train/test split.

Table 1: Full Effects Results. (*: best score)

Dataset	Scores
	**JNLPBA**: 81.10, **Linnaeus**: 83.28, **NCBI-Disease**: 83.72
BioNLP09	**AnatEM**: 83.24, **BC2GM**: 83.56, **BC4CHEMD**: 81.89, **BC5CDR**: 83.35, **BioNLP11EPI**: 84.14, **BioNLP11ID**: 83.50, **BioNLP13CG**: 83.68, **BioNLP13GE**: 84.16*, **BioNLP13PC**: 83.53, **CRAFT**: 82.97, **Ex-PTM**: 83.86, **JNLPBA**: 82.29, **Linnaeus**: 82.78, **NCBI-Disease**: 83.55
BioNLP11EPI	**AnatEM**: 76.62, **BC2GM**: 76.60, **BC4CHEMD**: 74.48, **BC5CDR**: 76.67, **BioNLP09**: 78.10*, **BioNLP11ID**: 76.86, **BioNLP13CG**: 76.97, **BioNLP13GE**: 77.49, **BioNLP13PC**: 77.14, **CRAFT**: 75.80, **Ex-PTM**: 77.99, **JNLPBA**: 74.87, **Linnaeus**: 76.62, **NCBI-Disease**: 76.51
BioNLP11ID	**AnatEM**: 81.43, **BC2GM**: 81.35, **BC4CHEMD**: 77.16, **BC5CDR**: 81.43, **BioNLP09**: 81.87, **BioNLP11EPI**: 81.76, **BioNLP13CG**: 81.90, **BioNLP13GE**: 82.26*, **BioNLP13PC**: 81.66, **CRAFT**: 80.36, **Ex-PTM**: 81.73, **JNLPBA**: 78.80, **Linnaeus**: 81.62, **NCBI-Disease**: 81.78
BioNLP13CG	**AnatEM**: 75.85, **BC2GM**: 73.94, **BC4CHEMD**: 68.73, **BC5CDR**: 76.05, **BioNLP09**: 75.41, **BioNLP11EPI**: 75.78, **BioNLP11ID**: 76.58, **BioNLP13GE**: 76.26, **BioNLP13PC**: 77.33*, **CRAFT**: 74.08, **Ex-PTM**: 77.16, **JNLPBA**: 70.46, **Linnaeus**: 75.09, **NCBI-Disease**: 75.72
BioNLP13GE	**AnatEM**: 74.05, **BC2GM**: 74.08, **BC4CHEMD**: 73.19, **BC5CDR**: 73.48, **BioNLP09**: 75.99, **BioNLP11EPI**: 76.09*, **BioNLP11ID**: 73.66, **BioNLP13CG**: 75.35, **BioNLP13PC**: 73.99, **CRAFT**: 75.46, **Ex-PTM**: 73.78, **JNLPBA**: 74.15, **Linnaeus**: 74.16, **NCBI-Disease**: 74.05
BioNLP13PC	**AnatEM**: 79.61, **BC2GM**: 77.78, **BC4CHEMD**: 75.72, **BC5CDR**: 79.79, **BioNLP09**: 79.08, **BioNLP11EPI**: 79.31, **BioNLP11ID**: 80.67, **BioNLP13CG**: 80.36, **BioNLP13GE**: 80.76, **CRAFT**: 77.66, **Ex-PTM**: 80.94*, **JNLPBA**: 78.73, **Linnaeus**: 78.60, **NCBI-Disease**: 79.55
CRAFT	**AnatEM**: 77.08, **BC2GM**: 76.97, **BC4CHEMD**: 73.61, **BC5CDR**: 77.97, **BioNLP09**: 77.70, **BioNLP11EPI**: 77.61, **BioNLP11ID**: 78.10, **BioNLP13CG**: 77.30, **BioNLP13GE**: 78.48*, **BioNLP13PC**: 77.93, **Ex-PTM**: 78.36, **JNLPBA**: 74.86, **Linnaeus**: 77.38, **NCBI-Disease**: 77.43
Ex-PTM	**AnatEM**: 68.45, **BC2GM**: 68.35, **BC4CHEMD**: 60.33, **BC5CDR**: 69.46, **BioNLP09**: 72.00, **BioNLP11EPI**: 73.58*, **BioNLP11ID**: 69.58, **BioNLP13CG**: 68.82, **BioNLP13GE**: 70.07, **BioNLP13PC**: 70.36, **CRAFT**: 67.25, **JNLPBA**: 62.60, **Linnaeus**: 69.20, **NCBI-Disease**: 68.49
JNLPBA	**AnatEM**: 68.19, **BC2GM**: 68.20, **BC4CHEMD**: 66.49, **BC5CDR**: 68.77, **BioNLP09**: 68.11, **BioNLP11EPI**: 68.33, **BioNLP11ID**: 68.19, **BioNLP13CG**: 68.54, **BioNLP13GE**: 68.92*, **BioNLP13PC**: 68.84, **CRAFT**: 67.97, **Ex-PTM**: 68.84, **Linnaeus**: 68.18, **NCBI-Disease**: 68.51
Linnaeus	**AnatEM**: 83.23, **BC2GM**: 81.71, **BC4CHEMD**: 79.24, **BC5CDR**: 82.83, **BioNLP09**: 83.12, **BioNLP11EPI**: 82.20, **BioNLP11ID**: 81.77, **BioNLP13CG**: 80.47, **BioNLP13GE**: 82.81, **BioNLP13PC**: 82.68, **CRAFT**: 81.21, **Ex-PTM**: 82.37, **JNLPBA**: 77.06, **NCBI-Disease**: 83.63*
NCBI-Disease	**AnatEM**: 79.76, **BC2GM**: 78.40, **BC4CHEMD**: 75.16, **BC5CDR**: 79.98, **BioNLP09**: 78.97, **BioNLP11EPI**: 79.75, **BioNLP11ID**: 79.24, **BioNLP13CG**: 79.85, **BioNLP13GE**: 80.06, **BioNLP13PC**: 79.41, **CRAFT**: 76.96, **Ex-PTM**: 80.74*, **JNLPBA**: 74.84, **Linnaeus**: 79.21

References

[Bada et al.2012] Michael Bada, Miriam Eckert, Donald Evans, Kristin Garcia, Krista Shipley, Dmitry Sitnikov, William A Baumgartner, K Bretonnel Cohen, Karin Verspoor, Judith A Blake, et al. 2012. Concept annotation in the CRAFT corpus. *BMC bioinformatics*, 13(1):1.

[Campos et al.2013] David Campos, Sérgio Matos, and José Luís Oliveira. 2013. Gimli: open source and high-performance biomedical name recognition. *BMC bioinformatics*, 14(1):54.

[Comeau et al.2013] Donald C Comeau, Rezarta Islamaj Doğan, Paolo Ciccarese, Kevin Bretonnel Cohen, Martin Krallinger, Florian Leitner, Zhiyong Lu, Yifan Peng, Fabio Rinaldi, Manabu Torii, et al. 2013. Bioc: a minimalist approach to interoperability for biomedical text processing. *Database*, 2013:bat064.

[Doğan et al.2014] Rezarta Islamaj Doğan, Robert Leaman, and Zhiyong Lu. 2014. NCBI disease corpus: a resource for disease name recognition and concept normalization. *Journal of biomedical informatics*, 47:1–10.

[Gerner et al.2010] Martin Gerner, Goran Nenadic, and Casey M Bergman. 2010. LINNAEUS: a species name identification system for biomedical literature. *BMC bioinformatics*, 11(1):1.

[Kim et al.2004] Jin-Dong Kim, Tomoko Ohta, Yoshimasa Tsuruoka, Yuka Tateisi, and Nigel Collier. 2004. Introduction to the bio-entity recognition task at JNLPBA. In *Proceedings of JNLPBA*, pages 70–75.

[Kim et al.2008] Jin-Dong Kim, Tomoko Ohta, and Jun'ichi Tsujii. 2008. Corpus annotation for mining biomedical events from literature. *BMC bioinformatics*, 9(1):1.

[Kim et al.2009] Jin-Dong Kim, Tomoko Ohta, Sampo Pyysalo, Yoshinobu Kano, and Jun'ichi Tsujii. 2009. Overview of bionlp'09 shared task on event extraction. In *Proceedings of the Workshop on Current Trends in Biomedical Natural Language Processing: Shared Task*, pages 1–9. Association for Computational Linguistics.

[Kim et al.2011] Jin-Dong Kim, Sampo Pyysalo, Tomoko Ohta, Robert Bossy, Ngan Nguyen, and Jun'ichi Tsujii. 2011. Overview of bionlp shared task 2011. In *Proceedings of the BioNLP Shared Task 2011 Workshop*, pages 1–6.

[Kim et al.2013] Jin-Dong Kim, Yue Wang, and Yamamoto Yasunori. 2013. The genia event extraction shared task, 2013 edition-overview. In *Proceedings of the BioNLP Shared Task 2013 Workshop*, pages 8–15. Association for Computational Linguistics.

[Krallinger et al.2015] Martin Krallinger, Florian Leitner, Obdulia Rabal, Miguel Vazquez, Julen Oyarzabal, and Alfonso Valencia. 2015. CHEMDNER: The drugs and chemical names extraction challenge. *J. Cheminformatics*, 7(S-1):S1.

[Leaman and Gonzalez2008] Robert Leaman and Graciela Gonzalez. 2008. BANNER: an executable survey of advances in biomedical named entity recognition. In *Proceedings of PSB*, volume 13, pages 652–663.

[Mi and Thomas2009] Huaiyu Mi and Paul Thomas. 2009. Panther pathway: an ontology-based pathway database coupled with data analysis tools. *Protein Networks and Pathway Analysis*, pages 123–140.

[Ogren2006] Philip V Ogren. 2006. Knowtator: a protégé plug-in for annotated corpus construction. In *Proceedings of NAACL-HTL 2006*, pages 273–275.

[Ohta et al.2002] Tomoko Ohta, Yuka Tateisi, and Jin-Dong Kim. 2002. The GENIA corpus: An annotated research abstract corpus in molecular biology domain. In *Proceedings of HTL*, pages 82–86.

[Ohta et al.2012] Tomoko Ohta, Sampo Pyysalo, and Sophia Ananiadou. 2012. Open-domain anatomical entity mention detection.

[Pyysalo and Ananiadou2013] Sampo Pyysalo and Sophia Ananiadou. 2013. Anatomical entity mention recognition at literature scale. *Bioinformatics*, 30:btt580.

[Pyysalo et al.2011] Sampo Pyysalo, Tomoko Ohta, Makoto Miwa, and Jun'ichi Tsujii. 2011. Towards exhaustive protein modification event extraction. In *Proceedings of BioNLP 2011 Workshop*, pages 114–123. Association for Computational Linguistics.

[Pyysalo et al.2012a] Sampo Pyysalo, Tomoko Ohta, Makoto Miwa, Han-Cheol Cho, Jun'ichi Tsujii, and Sophia Ananiadou. 2012a. Event extraction across multiple levels of biological organization. *Bioinformatics*, 28(18):i575–i581.

[Pyysalo et al.2012b] Sampo Pyysalo, Tomoko Ohta, Rafal Rak, Dan Sullivan, Chunhong Mao, Chunxia Wang, Bruno Sobral, Jun'ichi Tsujii, and Sophia Ananiadou. 2012b. Overview of the ID, EPI and REL tasks of BioNLP shared task 2011. *BMC bioinformatics*, 13(11):1.

[Sasaki et al.2008] Yutaka Sasaki, Yoshimasa Tsuruoka, John McNaught, and Sophia Ananiadou. 2008. How to make the most of ne dictionaries in statistical ner. *BMC bioinformatics*, 9(11):1.

[Settles2005] Burr Settles. 2005. ABNER: An open source tool for automatically tagging genes, proteins, and other entity names in text. *Bioinformatics*, 21(14):3191–3192.

[Smith et al.2008] Larry Smith, Lorraine K Tanabe, Rie Johnson nee Ando, Cheng-Ju Kuo, I-Fang Chung, Chun-Nan Hsu, Yu-Shi Lin, Roman Klinger, Christoph M Friedrich, Kuzman Ganchev, et al. 2008. Overview of BioCreative II gene mention recognition. *Genome biology*, 9(Suppl 2):1–19.

[Tanabe et al.2005] Lorraine Tanabe, Natalie Xie, Lynne H Thom, Wayne Matten, and W John Wilbur. 2005. Genetag: a tagged corpus for gene/protein named entity recognition. *BMC bioinformatics*, 6(1):1.

[Tsuruoka et al.2005] Yoshimasa Tsuruoka, Yuka Tateishi, Jin-Dong Kim, Tomoko Ohta, John McNaught, Sophia Ananiadou, and Junichi Tsujii. 2005. Developing a robust part-of-speech tagger for biomedical text. In *Proceedings of Panhellenic Conference on Informatics*, pages 382–392.

[Verspoor et al.2012] Karin Verspoor, Kevin Bretonnel Cohen, Arrick Lanfranchi, Colin Warner, Helen L Johnson, Christophe Roeder, Jinho D Choi, Christopher Funk, Yuriy Malenkiy, Miriam Eckert, et al. 2012. A corpus of full-text journal articles is a robust evaluation tool for revealing differences in performance of biomedical natural language processing tools. *BMC bioinformatics*, 13(1):1.

[Wei et al.2013] Chih-Hsuan Wei, Hung-Yu Kao, and Zhiyong Lu. 2013. PubTator: a web-based text mining tool for assisting biocuration. *Nucleic acids research*, page gkt441.

[Wei et al.2015] Chih-Hsuan Wei, Yifan Peng, Robert Leaman, Allan Peter Davis, Carolyn J Mattingly, Jiao Li, Thomas C Wiegers, and Zhiyong Lu. 2015. Overview of the Biocreative V chemical disease relation (CDR) task. In *Proceedings of the BioCreative 5 workshop*, pages 154–166.
